# Copper sulfide as the cation exchange template for synthesis of bimetallic catalysts for CO_2_ electroreduction†

**DOI:** 10.1039/d1ra03811g

**Published:** 2021-07-07

**Authors:** Jinghan Li, Junrui Li, Chaochao Dun, Wenshu Chen, Di Zhang, Jiajun Gu, Jeffrey J. Urban, Joel W. Ager

**Affiliations:** State Key Laboratory of Metal Matrix Composites, School of Materials Science and Engineering, Shanghai Jiao Tong University Shanghai 200240 China; Joint Center for Artificial Photosynthesis, Materials Sciences Division and Chemical Sciences Division, Lawrence Berkeley National Laboratory Berkeley CA 94720 USA jwager@lbl.gov; Department of Materials Science and Engineering, University of California Berkeley CA 94720 USA; The Molecular Foundry, Lawrence Berkeley National Laboratory Berkeley CA 94720 USA; The School of Environmental Science, Nanjing Key Laboratory of Advanced Functional Materials, Nanjing Xiaozhuang University Nanjing Jiangsu 211171 China

## Abstract

Among metals used for CO_2_ electroreduction in water, Cu appears to be unique in its ability to produce C2+ products like ethylene. Bimetallic combinations of Cu with other metals have been investigated with the goal of steering selectivity *via* creating a tandem pathway through the CO intermediate or by changing the surface electronic structure. Here, we demonstrate a facile cation exchange method to synthesize Ag/Cu electrocatalysts for CO_2_ reduction using Cu sulfides as a growth template. Beginning with Cu_2−*x*_S nanosheets (C-nano-0, 100 nm lateral dimension, 14 nm thick), varying the Ag^+^ concentration in the exchange solution produces a gradual change in crystal structure from Cu_7_S_4_ to Ag_2_S, as the Ag/Cu mass ratio varies from 0.3 to 25 (CA-nano-*x*, *x* indicating increasing Ag fraction). After cation exchange, the nanosheet morphology remains but with increased shape distortion as the Ag fraction is increased. Interestingly, the control (C-nano-0) and cation exchanged nanosheets have very high faradaic efficiency for producing formate at low overpotential (−0.2 V *vs.* RHE). The primary effect of Ag incorporation is increased production of C2+ products at −1.0 V *vs.* RHE compared with C-nano-0, which primarily produces formate. Cation exchange can also be used to modify the surface of Cu foils. A two-step electro-oxidation/sulfurization process was used to form Cu sulfides on Cu foil (C-foil-*x*) to a depth of a few 10 s of microns. With lower Ag^+^ concentrations, cation exchange produces uniformly dispersed Ag; however, at higher concentrations, Ag particles nucleate on the surface. During CO_2_ electroreduction testing, the product distribution for Ag/Cu sulfides on Cu foil (CA-foil-*x-y*) changes in time with an initial increase in ethylene and methane production followed by a decrease as more H_2_ is produced. The catalysts undergo a morphology evolution towards a nest-like structure which could be responsible for the change in selectivity. For cation-exchanged nanosheets (CA-nano-*x*), pre-reduction at negative potentials increases the CO_2_ reduction selectivity compared to tests of as-synthesized material, although this led to the aggregation of nanosheets into filaments. Both types of bimetallic catalysts are capable of selective reduction of CO_2_ to multi-carbon products, although the optimal configurations appear to be metastable.

## Abstract 2

Cu sulfides as a template for Ag/Cu sulfide catalysts for electrochemical CO_2_. With the introduction of Ag, nanosheet show increased C2+ product generation. The catalysts undergo a morphology evolution as CO_2_ reduction proceeds.

## Graphical abstract

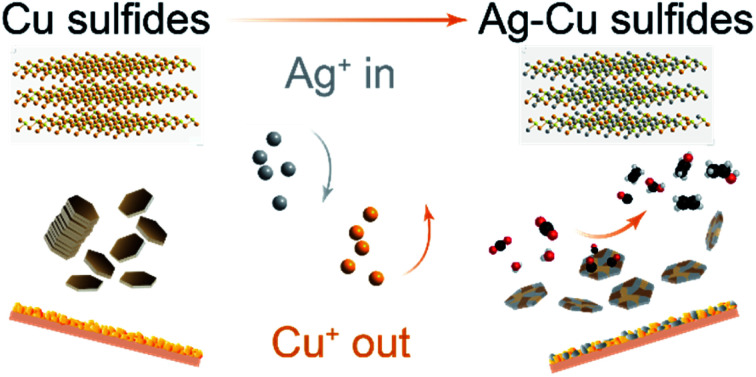

## Introduction

CO_2_ electroreduction (CO_2_R) has become one of the most promising strategies towards achieving a carbon-neutral environment. Provided that it is powered by a renewable energy source, it can sustainably convert the greenhouse gas CO_2_ into fuels like methanol and ethanol, and commodity chemicals such as ethylene.^1,2^ Cu has been of intense interest as an electrocatalyst for this reaction, as it is selective for CO_2_ reduction over water reduction and can produce C2+ products, due to its positive adsorption energy for H* and more optimal binding energy for CO_2_ and related intermediates, compared to other metals.^3–6^ Still, it has been difficult to control selectivity to a single CO_2_ reduction product. To this end, many research groups investigated alloys and bimetallic configurations of Cu with other metals to attempt to tune the overall catalyst performance.^3^

There are two main conceptual strategies for Cu-based alloy and bimetallic electrocatalysts. One approach is to create a tandem catalyst mechanism *via* combining Cu with other CO-producing elements like Ag or Au. In this concept, the crucial intermediate CO made on the second metal surface can transfer to Cu to be further reduced.^7–12^ For example, polycrystalline copper foil with Au nanoparticles favors the generation of oxygenates over hydrocarbons at low overpotentials.^13^ Increased CO concentration achieved by Ag nanoparticles on oxide-derived Cu nanowires may also open another pathway, namely *CO + *CH_*x*_ coupling towards increased ethanol generation.^14^ A second approach is to change the local electronic structure of Cu by alloying with the other elements in order to tune the binding strength towards intermediates.^15–19^ For example, Ag atoms in the bimetallic Cu–Ag catalyst create a diversity of binding configurations compared with pure Cu that facilitates the production of ethanol.^20^ The compressive surface strain induced by Ag reduces the H* adsorbates, leading to the selective suppression of HER and favors the production of multi-carbon oxygenates.^21^ Ag_2_Cu_2_O_3_, with a 1 : 1 stoichiometric ratio between Ag and Cu, can be used to produce bimetallic catalysts with a known composition and uniform distribution on the atomic scale. When applied to CO reduction, catalysts of this type can achieve 92% faradaic efficiency towards C2+ products at 600 mA cm^−2^.^22^

In this context, a facile strategy to introduce another element of specific concentration mixed with Cu on a variety of catalyst morphologies would be beneficial. This motivated us to investigate the cation exchange method, whereby a guest metal is introduced in the ion-form to replace the host metal ion in the compounds partially or entirely. This chemical conversion method has been widely employed to metal sulfides and oxides to achieve metastable facets, heteroatom doping, and introducing defect and strain, and also can be used to make multi-metal catalysts.^23^ Specific to CO_2_ reduction, the choice of the parent compound used for cation exchange may influence catalyst performance. We note here the reported enhancement in C2+ product selectivity for oxide-derived copper as compared to metallic copper, some of which has been attributed to increased roughness and grain boundaries which form as a result of *in situ* reduction of the oxide starting material.^24–27^ Bearing this in mind, we hypothesized that Cu sulfide could serve as a convenient cation exchange template for the formation of bimetallic CO_2_R electrocatalysts. We further hypothesized that the depletion of sulfur which occurs at the negative potential used to drive CO_2_R could lead to morphology changes which could be beneficial for control of selectivity.^11,28^

To test these hypotheses, we developed a cation exchange method to accommodate two common catalyst designs for CO_2_ reduction: nanoparticles and foil electrodes. Ag was selected as the second metal as it is selective for the production of CO, which is believed to be the key intermediate for the formation of C2+ products. For creating nanoscale catalysts, we prepared Cu sulfide nanosheets (C-nano-0) by colloidal synthesis, while for modifying Cu surfaces, Cu sulfides were directly grown on Cu foil using electro-oxidation followed by sulfurization. In both cases, the Ag/Cu mass ratio of the catalysts could be controlled at the cation exchange step. For nanosheets, the Ag/Cu mass ratio can reach 25 with the original structure remaining nearly intact. Cation exchange on surface-modified Cu foils (C-foil-*x*) produces well-dispersed Ag at low concentrations but leads to Ag particle nucleation at higher concentrations. Compared with C-nano-0 controls, CO_2_ reduction on moderately cation exchanged Ag/Cu sulfide nanosheets (CA-nano-2) increases the selectivity to C2+ products at −1.0 V *vs.* RHE. The selectivity for CO_2_ reduction of cation exchanged foils increases and then decreases over a period of 16 hours. Both the nanosheets and copper foil catalysts undergo noticeable morphology changes during the CO_2_ reduction, which may explain why the product distributions change as the CO_2_ reduction proceeds.

## Synthesis procedures

The synthesis methods are summarized here; full details are in ESI.†

### Cu sulfide nanosheets (C-nano-0)

**Scheme 1:**
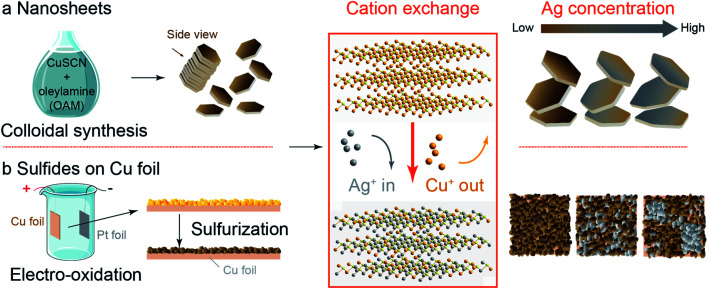
Synthetic strategies for Ag/Cu sulfide catalysts. (a) Cu sulfide nanosheets (C-nano-0, 100 nm lateral dimension, 14 nm thick) were obtained through colloidal synthesis with CuSCN in oleylamine (OAM). (b) Cu sulfides on Cu foil (C-foil-*x*) were obtained through electro-oxidation in 1 M NaOH to produce an oxide layer of a few 10 s of microns thick followed by sulfurization with 0.1 M Na_2_S. After cation exchange where Ag^+^ replaces the Cu^+^ in the Cu sulfides, Ag/Cu sulfide nanosheets (CA-nano-*x*) remain nanosheet structure with some distortion in shape as the Ag/Cu mass ratio ranges from 0.3 to 25; while for C-foil-*x*, Ag nucleates at higher Ag concentration, that impedes the uniform distribution of Ag and Cu.

C-nano-0 was synthesized with a modified colloidal synthesis recipe (Scheme 1a, see also Table 1 for sample nomenclature).^29,30^ Typically, 257 mg copper(i) thiocyanate (CuSCN) was dispersed in 25 mL oleylamine (OAM). The mixture was first degassed and heated in N_2_ to 240 °C for 30 min. The synthesized nanosheets were then washed with hexanes and ethanol to remove the surface ligands and dispersed in hexanes for storage.

**Table 1:** Cation-exchanged nanosheet samples with precursor and reagent contents and mass ratios as measured by inductively coupled plasma mass spectroscopy (ICP-MS)

Label	C-nano-0 (mg)	AgNO_3_ (mg)	Ag/Cu mass ratio
C-nano-0	—	0	—
CA-nano-1	30	15	0.3
CA-nano-2	30	30	0.8
CA-nano-3	30	45	2.3
CA-nano-4	30	90	25

### Cu sulfides on Cu foil (C-foil-*x*)

Cu sulfides on Cu foil (C-foil-*x*, see Table 2 for sample nomenclature) were synthesized with a two-step electro-oxidation/sulfurization process (Scheme 1b). Cu foil was first cleaned and etched by 4 M HCl. After that, Cu(OH)_2_ was grown on the Cu foil by electro-oxidation in 1 M NaOH. The electrode was then immersed in 0.1 M Na_2_S at 90 °C for 12 h to obtain Cu sulfides.^31^ The current density set during the electro-oxidation process affects the grain size, as will be discussed later. Carbon substrates including carbon paper and carbon cloth with deposited Cu as the Cu source and chemical oxidation for the growth of Cu(OH)_2_ were also tried but were less successful; see ESI for details (Fig. S1–S6†).

**Table 2:** Sulfides on Cu foil with electro-oxidation and cation exchange parameters

Label	Current density (mA cm^−2^)	AgNO_3_ (mg)
C-foil-10	10	—
C-foil-20	20	—
C-foil-30	30	—
C-foil-40	40	—
CA-foil-20-10	20	10
CA-foil-20-20	20	20
CA-foil-20-30	20	30
CA-foil-20-40	20	40
CA-foil-40-40	40	40

### Cation exchange method

For nanosheet samples, the hexanes dispersion containing C-nano-0 was added to an OAM solution (7 mL) containing the Ag precursor AgNO_3_.^29^ The solution was first degassed and heated to 50 °C in N_2_ and kept for another 30 min to complete the cation exchange reaction. The nanosheets were then washed with ethanol and hexanes and dispersed in hexanes for storage. The samples are denoted CA-nano-*x* as shown in Table 1.

To perform cation exchange for the Cu sulfides on Cu foil (C-foil-*x*), AgNO_3_ was added to OAM with N_2_ bubbled to the solution in small Petri-dish. After the solution was heated to 50 °C and well mixed, C-foil-*x* was placed in the solution and kept for another 30 min. The electrode was then cleaned with ethanol and hexanes and dried with N_2_ flow. The Ag/Cu sulfides on Cu foil were named CA-foil-*x-y* with *x* denoting the current density and *y* the relative Ag fraction (Table 2). As discussed later, the concentration of Ag^+^ in the cation exchange solution affected the dispersion of Ag, with uniform distributions being formed at low concentrations and Ag particles nucleating on the surface at high concentrations.

## Electrochemical characterization and product analysis

### Electrode preparation and CO_2_ reduction

For nanosheet samples (C-nano-0 and CA-nano-*x*), the catalysts were first anchored on carbon black at a 1 : 1 mass ratio of catalyst to carbon. The catalyst was dispersed in ethanol and water, and Nafion was added as the binder. After sonication, the homogeneous catalyst ink was drop cast on glassy carbon substrates (GC) followed by drying overnight at room temperature. Typically, the catalysts were tested with the loading of 0.6 mg on an electrode area of 0.785 cm^2^. Sulfides on Cu foil (C-foil-*x* and CA-foil-*x-y*) were tested directly without further modification. The CO_2_ reduction was conducted in a three-electrode system with 0.05 M K_2_CO_3_ as the electrolyte and Pt and Ag/AgCl (saturated KCl) as the reference and counter electrode, respectively. The cathodic and anodic chambers were separated by an anion exchange membrane. CO_2_ was purged at 5 sccm to the cathodic chamber, and the test started after 15 min CO_2_ purging to ensure complete saturation, after which the electrolyte becomes 0.1 M bicarbonate. We note that the freshly prepared electrodes are not active for CO_2_R and initially favour H_2_ production. The time evolution of the catalysts and the induced changes in the product profiles under CO_2_ electroreduction conditions are thus discussed in detail below.

### Product detection

The gas products, including H_2_, CO, methane, ethylene, and ethane, were detected by online gas chromatography (GC) using methods reported previously.^32,33^ Typically, GC sampling was started 5 min after the test began, and the results were given by the average of the second to the last sample. The electrolyte was collected after each test and analysed by nuclear magnetic resonance (NMR) for liquid products, including formate, methanol, ethanol, *n*-propanol, and other low-concentration C2+ products such as acetate, glycolaldehyde, allyl alcohol, acetaldehyde, acetone, and propionaldehyde.^6^

## Results and discussion

### Cu sulfide nanosheets (C-nano-0)

**Fig. 1:**
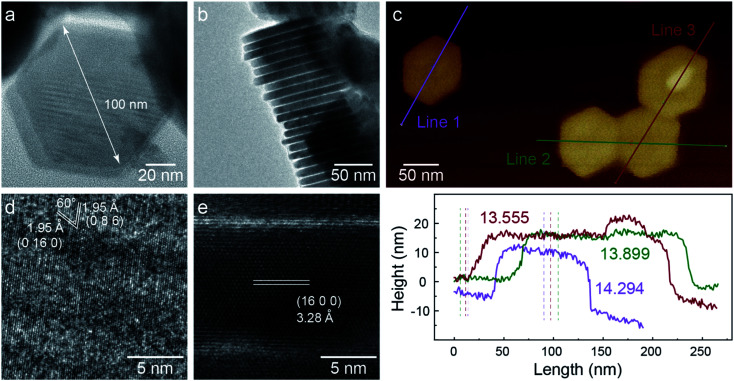
TEM characterization of C-nano-0. (a) C-nano-0 has a hexagonal shape with a lateral size of 100 nm. (b) Side-view of a stack of nanosheets. (c) AFM image and height information, showing the thickness of 14 nm. (d and e) High-resolution TEM (HRTEM) image shows the lattice structure of monoclinic Cu_7_S_4_.

TEM images of C-nano-0 made by colloidal synthesis are presented in Fig. 1. The nanosheets have a hexagonal shape with a lateral size of 100 nm and a thickness of 14 nm, as shown in Fig. 1a–c. As shown in the HRTEM image (Fig. 1d), the planes with the spacing of 1.95 Å and a 60° angle in between can be assigned to the (0 16 0) and (0 8 6) planes of monoclinic Cu_7_S_4_.^34^ In addition, the 3.28 Å lattice spacing in the side view HRTEM image matches the (16 0 0) plane (Fig. 1e). Some areas exhibit less contrast with no clear lattice; these areas may have a high defect concentration or be amorphous. The nanosheets have a large specific surface area which could be beneficial for catalytic activity.

XPS spectra of C-nano-0 provide information about the surface condition of the nanosheets (Fig. S7 and S8†). The S/Cu atomic ratio was 0.67, slightly higher than the stoichiometric ratio of Cu_7_S_4_, supporting the Cu_7_S_4_ lattice structure with a sulfur-rich surface of C-nano-0. Most Cu in the nanosheets has the valence state of 1+, the deviation of the spectrum may result from the defects in the nanosheets and Cu^2+^.^35,36^ The existence of N, the peaks at 163 eV in the S_2p_ spectrum, and the peak at 286 eV in the C_1s_ spectrum indicate the presence of a small amount of residual ligand –SCN from the precursor CuSCN.^37^ It is also possible that OAM is present despite the washing steps designs to remove it; however, prior studies have shown that it does not block active sites for CO_2_ electroreduction.^38^

### Bimetallic sulfide nanosheets (CA-nano-*x*)

**Fig. 2:**
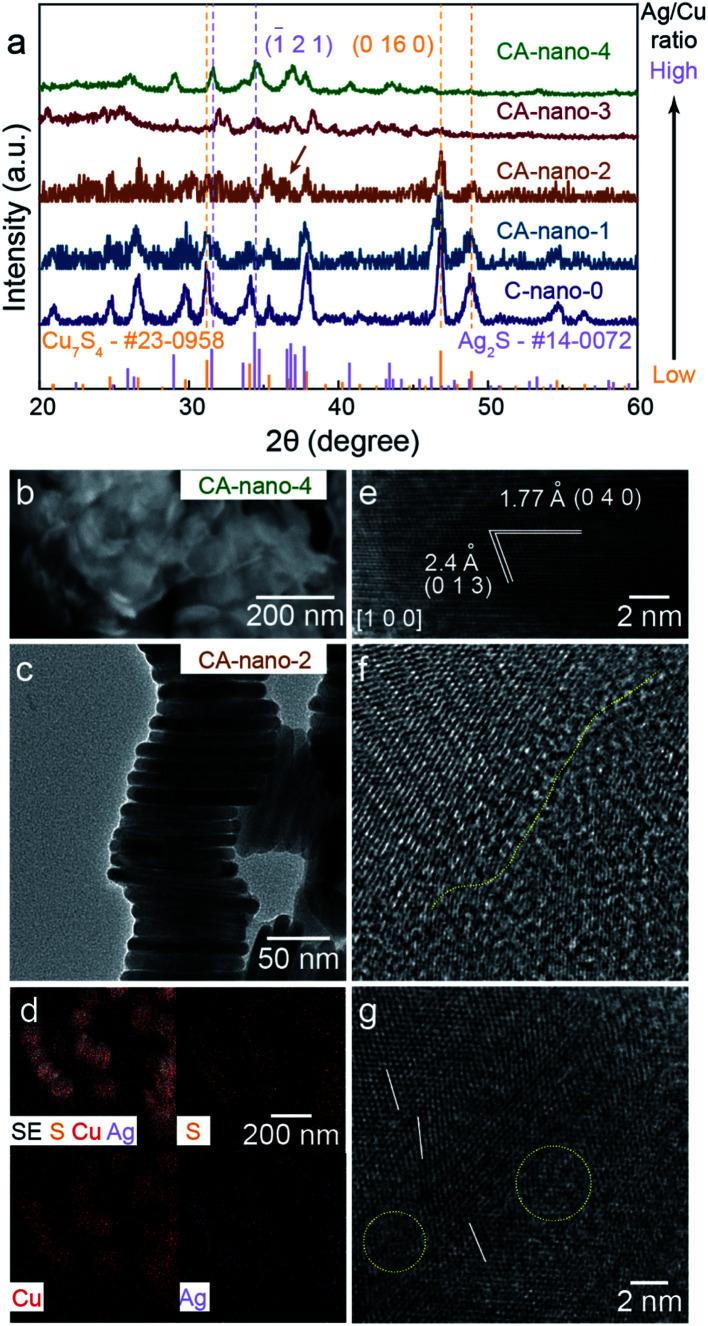
Lattice structure, morphology, and composition of sulfide nanosheets. (a) XRD spectra for C-nano-0 and CA-nano-*x*. Lattice structure evolves from Cu_7_S_4_ to Ag_2_S with increased Ag concentration. (b) SEM image for CA-nano-4, nanosheet structure remains with shape distortion. (c) Side-view TEM of stacked CA-nano-2, morphology is also shown in Fig. S13a.† (d) SEM and EDS mapping shows uniform distribution of Cu, Ag, and S within the nanosheets. (e) HRTEM image of CA-nano-2 shows the lattice structure of Ag_2_S viewed from direction [1 0 0]. (f and g) HRTEM images for the basal surface. Yellow and white marks denote the defect and boundary-rich surface.

The mass ratio of Ag/Cu concentration was well controlled from 0.3 (CA-nano-1) to 25 (CA-nano-4) (Table S1†). The ratio of Ag and Cu in samples were quantified by inductively coupled plasma mass spectroscopy (7900 ICP-MS, Agilent, ICP) using the He mode. The internal standard was Ge or Rh selected based on its first ionization potential and *M*/*Z* as compared to Cu or Ag, respectively. As shown in the XRD spectra (Fig. 2a), the crystal structure of the nanosheets undergoes a noticeable change as the Ag concentration increases. Prior to cation exchange, the nanosheets have the crystal structure of Cu_7_S_4_ (PDF #23-0958),^39^ in agreement with TEM. For a small amount of cation exchange (CA-nano-1), the crystal structure remains the same as the C-nano-0 with the most prominent peak in the XRD pattern at ∼48° being assigned to Cu_7_S_4_ (0 16 0). At higher Ag concentrations, a shift of the peak near ∼32° is observed from the yellow marked position for Cu_7_S_4_ to the purple marked Ag_2_S position. The appearance (CA-nano-2) and shift of the peak pointed by the arrow as Ag concentration increases support the gradual structural change. With the ratio of Ag/Cu reaches 25 (CA-nano-4), the structure completely changes to Ag_2_S, demonstrated by the peak at ∼34°, which can be assigned to (1̄ 2 1) planes. The morphology also undergoes obvious change with large shape distortion and the nanosheet structure remains (Fig. 2b), while with Ag/Cu ratio less than 1, the hexagonal shape remains with only minor changes in shape or thickness (Fig. S9–S12†).

Fig. 2c–g show the morphology, elemental distribution, and typical lattice structure of CA-nano-2 (Ag/Cu = 0.8) as determined by SEM and TEM. The morphology uniformity of the nanosheets decreases compared with that before cation exchange but the enlarged SEM image with elemental mapping still provides evidence of the uniform distribution of Ag, Cu, and S, without any spatial separation (Fig. 2d).

HRTEM images obtained from the side-view demonstrate the existence of crystalline Ag_2_S. In Fig. 2e, observed from direction [1 0 0], the plane with the spacing of 1.77 Å and 2.4 Å can be assigned to the planes (0 4 0) and (0 1 3), respectively. Besides, it shows a composition of small crystals with different facets on the basal surface (Fig. 2f and g). The yellow line marks the boundary between two crystals that possess different structures, the white lines show the tilt of the lattice, and the yellow circles mark less-contrast areas indicating defect and amorphous regions. Combined with HRTEM images showing different lattices and the corresponding FFT patterns obtained from CA-nano-2 (Fig. S13†), one conclusion can be made is that crystal structure becomes more complicated due to the introduction of Ag and that the cation exchange does not have a simple outcome, a single crystal Ag_2_S or dominant exposed facets, for instance.

The complicated surface outcome from cation exchange might arise from a number of factors: the hexagonal shape of C-nano-0 triggers cation exchange from the corners and form separate grains connected by grain boundaries; the intrinsic poor crystallinity of the template leads to inconsistent reaction tendencies at different areas; the energy imposed by the low temperature (50 °C) for cation exchange is not enough for atoms to move towards the more crystalline structure.^40^ Therefore, as expected, CA-nano-2 has a complicated defect and boundary-rich structure.

### Sulfide nanosheets for CO_2_ reduction

**Fig. 3:**
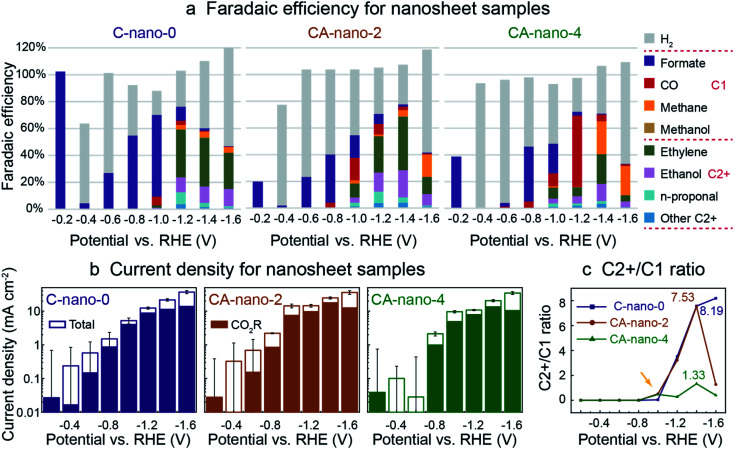
CO_2_ reduction performance for sulfide nanosheets: C-nano-0, CA-nano-2, and CA-nano-4. (a) Plot of faradaic efficiency (FE); note that all produce formate exclusively at low overpotentials (−0.2 V *vs.* RHE). (b) Total current density, solid portion of bar plot indicates partial current density for CO_2_ reduction products. Error bars of total current density at small negative potentials are relatively large as the current was fluctuating in time. The corresponding chronoamperometry (CA) plots are in Fig. S16.† (c) Plot for the ratio of C2+ to C1 products. Ag increases C2+ product generation at small negative potentials (−1.0 V *vs.* RHE). The numbers in the plot stand for the highest C2+/C1 ratio obtained for each catalyst. CO_2_ reduction was conducted in 0.05 M K_2_CO_3_ in an experimental sequence from more positive to more negative potentials. Catalyst loading was 0.6 mg in all cases.

SEM analysis shows that the nanosheets were evenly dispersed on porous carbon before CO_2_R (Fig. S14†). Prior *in situ* work with copper oxide pre-catalysts has shown that under CO_2_R conditions, reduction of oxides to metallic Cu occurs prior to the formation of gas phase products.^41^ We thus expected that sulfide nanosheets could have a similar behaviour, with the initial current being due to non-faradaic processes as the catalyst is reduced. Therefore, pre-reduction at negative potentials may increase selectivity for CO_2_ reduction. Additionally, the pre-reduction may facilitate the removal of the surface ligands –SCN, which might block or change the activity of the catalytic sites. Previous research show such anionic ligands could be removed under negative potentials and may induce reconstruction of the nano-scale catalysts, which further influence the performance.^42,43^ Thus, for a consistent comparison, the electrodes were evaluated in the same potential sequence. All samples were tested for the same 1.5 h duration from the most positive potential (−0.2 V *vs.* RHE) to the most negative potential (−1.6 V *vs.* RHE), as shown in Fig. 3 where two different cation-exchanged samples CA-nano-2 and CA-nano-4 are evaluated and compared with C-nano-0 control. Cyclic voltammetry (CV) measurements were conducted for the electrodes to show both Ag and Cu in the nanosheets are electrochemical active (Fig. S15†).^17^ It is worth mentioning that the current densities between the three samples are of similar values and trends, such that all catalysts had similar mass transfer limits for CO_2_ availability at a given potential (Fig. S16†). The electrochemical impedance spectra (EIS) indicate a lower ion transport resistance for cation exchanged samples (CA-nano-2 and CA-nano-4) compared with Cu sulfides (C-nano-0) (Fig. S17†).

Interestingly, at −0.2 V *vs.* RHE, Fig. 3a, all nanosheet catalysts produced formate exclusively before the production of other potential 2e^−^ products: CO and by-product H_2_ from HER. However, we note that the faradaic efficiency at −0.2 V *vs.* RHE cannot be measured precisely since the potentiostat current at this potential, ∼0.01 mA, is very small. Also, the larger current measured at the beginning of a run due to the non-faradic reduction of the catalyst surface sulfide or oxide layers, where not all the electrons were used for the formation of electrocatalytic CO_2_ reduction products, can lead to inaccuracy, especially for small negative potential regions, where large current fluctuations were observed as shown by the error bars (Fig. 3b). To assess whether formate was made just at the start or throughout the run, we tested CA-nano-2 again at −0.2 V *vs.* RHE after the electrode has been tested at more negative potentials (Fig. S18†). Formate was still the only product detected, although the initial current density was smaller compared with as-synthesized material. One explanation is the initial current was from the reduction of the surface oxidation layer formed in the environment after the previous test instead of the reduction of the catalyst as for fresh electrode.^44^ Formate was also the only product detected at −0.1 V and −0.3 V *vs.* RHE for nanosheets (Fig. S19†).

For all nanosheets, H_2_ appears as a product at −0.4 V *vs.* RHE. At larger negative potentials its FE decreases and FE for formate increases. For C-nano-0, CO appears at −1.0 V *vs.* RHE (FE_CO_ = 6%). C2+ products, including ethylene, ethanol, and C3 products like *n*-propanol, appear at −1.0 V *vs.* RHE in trace amounts and dominate at −1.2 V *vs.* RHE with the ratio of C2+ to C1 products of 3.51. This ratio further increases when a more negative potential is applied, 7.57 and 8.19 for −1.4 V and −1.6 V *vs.* RHE, respectively (Fig. 3c). However, this increase comes from the decrease of formate and methane, rather than increased production in C2+ products, and an increase in H_2_ production is also clearly shown since −1.0 V *vs.* RHE (Fig. S20†).

CA-nano-2 starts to produce noticeable C2+ products, including ethylene (10%), ethanol (4%), and *n*-propanol (3%) at −1.0 V *vs.* RHE, more positive than for C-nano-0. Also, the overall CO_2_ reduction products dominate at −1.4 V *vs.* RHE (77.7%), which is shifted from −1.2 V for C-nano-0; this may be attributed to better HER suppression at more negative potentials as a result of the Ag content.^18^ For CA-nano-2 at −1.4 V *vs.* RHE, the faradaic efficiency for C2+ products is 68.6%, and the ratio of C2+ over C1 products is 7.53. The C2+/C1 ratio is similar to that of the control (Fig. 3c), but with a smaller FE for H_2_. The introduction of Ag leads to an increase in CO production compare to the control, with the maximum FE reached −1.0 V *vs.* RHE. When a more negative potential is applied, the FE for CO decreases, which could be a result of CO diffusion to Cu where it is further reduced to C2+ products.^8^ At −1.6 V *vs.* RHE, where Ag has less contribution to reducing CO_2_ to CO but instead increases HER, methane becomes the dominant product.^45^

For CA-nano-4, where Ag/Cu ratio is 25, CO production from Ag becomes the primary product, especially at −1.2 V *vs.* RHE (53%), much higher than that of CA-nano-2 (8%). Also, methane is a dominant product at negative potentials (25% at −1.4 V *vs.* RHE). One explanation for the large increase in C1 products might be that the increased concentration of Ag breaks up the continuous Cu surface, making the CO–CO binding difficult.^46^ Thus, instead of making C2 products like ethylene and ethanol, conversion of the CO intermediate forms methane. Indeed, for this catalyst, the highest C2+/C1 ratio was only 1.33 (Fig. 3c). Similar to the other nanosheet samples, with more negative potential applied, H_2_ became the dominant product, which also caused a decrease in the current density for overall CO_2_ reduction at −1.6 V *vs.* RHE where more surface has been occupied by the H absorbent for H_2_, instead of performing CO_2_ reduction.

### Sulfides on Cu foil. (C-foil-*x* and CA-foil-*x-y*)

**Fig. 4:**
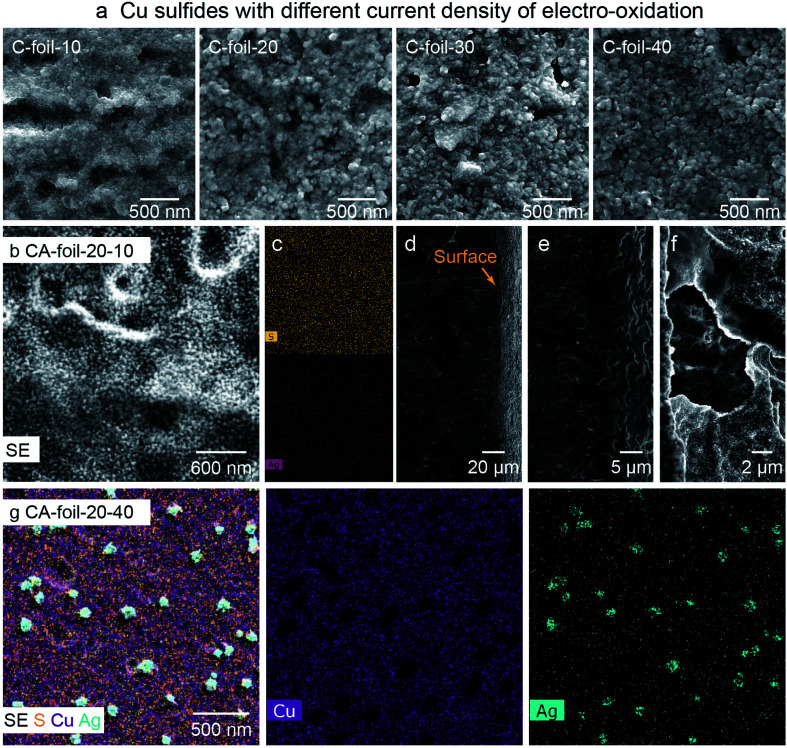
Morphology characterization for sulfides on Cu foil. (a) SEM images for Cu sulfides (C-foil-*x*) obtained from the sulfurization of Cu(OH)_2_ achieved by electro-oxidation at the current density of 10, 20, 30, and 40 mA cm^−2^. Morphology (b) and elemental distribution (c) from top view and side view (d–f) of bimetallic sulfides on Cu foil (CA-foil-*x-y*) with moderate Ag concentration and extra Ag concentration (g).

For bimetallic sulfides on Cu foil, the crystal size of the Cu sulfide template (C-foil-*x*, *x* denoting current density, Table 2) can be controlled by the current density during the electro-oxidation process. Larger and more defined grains are produced at higher current densities (Fig. 4a). For cation-exchanged samples (CA-foil-*x-y*), the Ag/Cu mass ratio increases as more Ag precursor is added during the cation exchange (Fig. S21†). With lower Ag concentrations (CA-foil-20-10), the Ag and S were uniformly distributed across the surface (Fig. 4b and c). SEM image from the side view indicates the thickness of 10 s of microns, consistent with the calculated modified Cu thickness (Fig. 4d, ESI†). Also, compared with C-foil-20, the surface of the cation-exchanged counterpart CA-foil-20-10 transformed into a rippled structure composed of finer grains, with empty spaces between layers, demonstrated by zoomed-in images, that might be beneficial for the transport of reactants and products (Fig. 4e and f). However, with further increased Ag precursor AgNO_3_, Ag nucleation on the surface through the reduction of Ag^+^ to metallic Ag instead of exchanging Cu^+^ can be observed (CA-foil-20-40). Ag concentration at the newly merged flower-like flakes is clearly shown in Fig. 4g and S22.† When the particle size of Cu sulfides was too large (C-foil-40), the cation-exchanged counterpart CA-foil-40-40 with enough Ag precursor (40 mg) grow into a triangle structure (Fig. S23†).

### Evolution of sulfide catalysts during the CO_2_ reduction

**Fig. 5:**
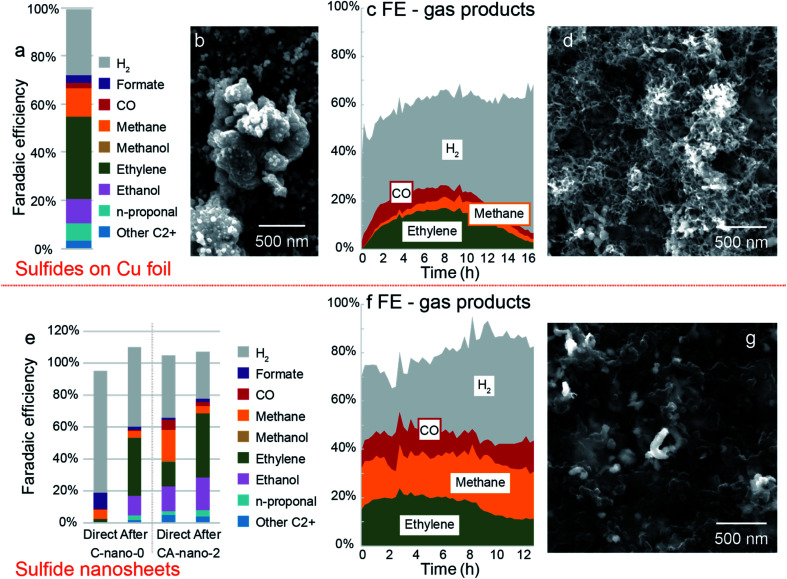
Evolution of catalysts and corresponding products change during CO_2_ reduction. (a) Faradaic efficiency plot for CA-foil-20-40 at −1.2 V *vs.* RHE. (b) Morphology of CA-foil-20-40 after CO_2_ reduction. Corresponding current plot is in Fig. S24.† (c) Faradaic efficiency for gas products. Sample: CA-foil-20-40. Test condition: −1.0 V *vs.* RHE for 16 h. Corresponding current plot is in Fig. S26.† (d) SEM image of CA-foil-20-40 after test. (e) Comparison of sulfide nanosheet C-nano-0 and CA-nano-2 between directly tested at −1.4 V *vs.* RHE (left bar plot) and tested after pre-reduction at other negative potentials (right bar plot). (f) Faradaic efficiency for gas products. Sample: CA-nano-4. Test condition: −1.4 V *vs.* RHE for 13 h. Corresponding current plot is in Fig. S29.† (g) SEM image of CA-nano-4 after test.

Bimetallic sulfides on Cu foil (CA-foil-*x-y*) with a variety of Ag concentrations were tested in the potential range from −0.8 V to −1.4 V *vs.* RHE. If considering ethylene as the target product, CA-foil-20-40 with high surface Ag concentration at −1.2 V *vs.* RHE gave the best performance (FE_ethylene_ = 34%, Fig. 5a and S24†). However, results of different runs show large variations in terms of both FE and current density, with selectivity to ethylene and liquid C2+ products initially improving and then declining (3 tests at −1.0 V *vs.* RHE and then 3 at −1.2 V *vs.* RHE, Fig. S25†). The morphology after reduction also shows obvious change from the fresh sample, which may come from the reaction between the active surface and the reactant and intermediates (Fig. 5b).

To further investigate this performance change, as-synthesized CA-foil-20-40 was directly tested at −1.0 V *vs.* RHE for 16 h. The current was in the range of 8–12 mA cm^−2^ during the test, Fig. S26.† Faradaic efficiency for the gas products is plotted in Fig. 5c. CO was the first gas product of CO_2_ reduction to be observed and reaches a maximum in FE at 2 hours. Ethylene and methane increased with the consumption of CO, and FE for ethylene rose from 0% at first to 17% at 8 h. As the plot shows, the product profile undergoes continuous change during CO_2_ reduction, with the optimal working region for ethylene being between 6 to 10 h.

The evolution of the catalyst surface may explain the performance change.^18,47^Fig. 5d is the SEM image of the catalyst after the 16 h reduction. The surface morphology changes from the particle shape into the nest structure composed of filaments with a diameter of ∼20 nm, which appears to have a higher surface area compared with the fresh sample. The increased roughness of the catalyst during the CO_2_ reduction might be one reason for the change of gas product distribution since the nest composed of the 1D filament structure possesses both high surface area and good electrical conductivity derived from the interconnected nature and structural stability compared with nanoparticle counterparts.^48^

C-foil-*x* also undergoes similar morphology change under CO_2_R conditions, with smaller grains (C-foil-10), the surface evolved into a nest structure composed of filaments (diameter: 40 nm). The time evolution of the catalyst is also influenced by the particle size of Cu sulfides on Cu foil, as the grain size increases as a higher current applied in the electro-oxidation step, the nest structure evolved from CO_2_R that possess high roughness and sufficient gas pathway, which might be beneficial, gradually disappears and is replaced by large particles (Fig. S27†). According to the research on oxide-derived copper. The surface copper oxide may be reduced to metallic copper under negative potential, while other groups have reported that the residual underlying oxygen is beneficial for CO_2_ reduction.^25,49^ The sulfides on Cu foil may also go through a similar process as Cu oxides, and the sulfur will eventually be depleted such that the catalyst will be metallic copper; thus the depletion of sulfur may result in the evolution of morphology and performance.^50^

Besides, as previously mentioned, for sulfides on the Cu foil electrode, there is a limit for the introduction of Ag because the cation exchange of Cu^+^ by Ag^+^ competes with the direct nucleation on the electrode surface. Thus, in order to create an Ag-rich surface by other means, we deposited 100 nm Ag by an E-beam evaporator onto the Cu sulfides layer. However, when tested at −1.1 V *vs.* RHE, the potential most beneficial for Ag foil to produce CO,^51^ the morphology change from the flat overlayer to more complex nest structures during the CO_2_ reduction still occurred and exposed the underlying Cu sites. As a result, the product distribution was very different from that expected from a silver-rich surface^52^ (Fig. S28†).

### Activation of cation-exchanged nanosheets

For CA-nano-*x* nanosheets, when put directly under CO_2_ reduction conditions, the performance is far from ideal. Thus we tried to employ pre-reduction under other negative potentials as an activation method to improve CO_2_R selectivity. As shown in Fig. 5e, freshly prepared C-nano-0 and CA-nano-2 were directly placed at −1.4 V *vs.* RHE, and the results are compared with that after pre-reduction at other negative potentials. For direct, much more H_2_ was produced compared with the pre-reduced electrode results, the FE for H_2_ was 76% for C-nano-0 and 39% for CA-nano-2, while for pre-reduced samples, the number was only 50% and 30%, respectively. The product distribution of CO_2_ reduction also varies from the pre-reduced electrodes. For example, for fresh C-nano-0, the major CO_2_ reduction products are C1 species, including formate (10%) and methane (6%). In contrast, when the sample was tested at the same potential after pre-reduction, there were mainly C2+ products. The situation was similar for cation exchanged nanosheets. The total FE for C2+ products for fresh CA-nano-2 was 38.02%, only half the C2+ products observed for the pre-reduced electrode (69%).

After CO_2_ reduction, as expected, the adjacent nanosheets were evolved into a filament structure with a diameter of ∼100 nm, similar to the diameter of the synthesized nanosheets, and the product profile changes accordingly (Fig. 5f and g). The change might be related to the surface ligands, which may affect electrochemical behavior and the stability of nanomaterials, yet more characterizations need to be done to confirm this.^42,53,54^ Additionally, for C-nano-0, the Cu was transformed into large clusters (∼500 nm, Fig. S30†), different from the filament structure of CA-nano-2. This might come from the difference in the CO/CO_2_ binding energy on Cu and Ag.^55^ With higher binding energy between the Cu and CO/CO_2_ facilitate the mobility of the anchored Cu, thus resulting in the aggregation. However, the Ag added tothe CA-nano-2 has smaller binding energy with CO/CO_2_ and confined the mobility, and the spatial separation of the nanosheets prevents further agglomeration.

## Conclusions

We have demonstrated that Cu sulfides can be used as a template for cation exchange to achieve bimetallic Ag/Cu sulfide catalysts with a well-controlled Ag/Cu mass ratio by changing the concentration of Ag precursor AgNO_3_. For nanosheets, the Ag/Cu ratio can reach 25 with the nanosheet structure remaining, while it is difficult to produce an Ag-rich surface beginning with sulfides on Cu foil. Formate was the only product detected at low overpotentials (−0.2 V *vs.* RHE), and with the introduction of moderate Ag, nanosheet catalysts showed increased C2+ product generation for CO_2_ reduction. The product profiles appear to be influenced by CO availability controlled by Ag concentration, suggesting a possible tandem catalytic mechanism. The reconstruction of the catalyst during CO_2_ reduction increased the production of multi-carbon products.

The cation exchange method can be further applied to other bimetallic or trimetallic chalcogenides like phase segregated Cu–Au sulfides,^40^ Cu–Ni selenides,^56^ Cu–Co sulfides nanoboxes,^57^ and CuInS_2_-doped ZnS,^58^ and could potentially be used for multifunctional photo/electrocatalysis. With modifications of ligands or additives during the cation exchange method, may realize the control of even *vs.* uneven distribution of two elements with the same overall concentration that can be employed as a great test field for mechanism investigation.

## Author contributions

Jinghan Li and J. W. A. conceptualized the study, and JWA acquired funding for it. Jinghan Li and Junrui Li developed synthesis methodology and analytical chemistry methods. Jinghan Li performed all synthesis and conducted the electrochemical experiments, XRD, XPS, ICP, AFM, and SEM characterization and data interpretation. Chaochao Dun performed TEM characterization using funding acquired by Jeffrey J. Urban. Wenshu Chen performed synthesis. Di Zhang and Jiajun Gu provided advice on data analysis and mentorship. Jinghan Li and JWA wrote the original draft of the manuscript and all authors contributed to its final editing.

## Conflicts of interest

There are no conflicts to declare.

## Supplementary Material

RA-011-D1RA03811G-s001
