# Supplementary material for: Copper sulfide as the cation exchange template for synthesis of bimetallic catalysts for CO2 electroreduction
Source: RSC Adv. 2021 Jul 7;11(39):23948–59. doi: 10.1039/d1ra03811g (PMC9036827; doi:10.1039/d1ra03811g)
Supplement: RA-011-D1RA03811G-s001 [file RA-011-D1RA03811G-s001.pdf]

Electronic Supplementary Material (ESI) for RSC Advances.
This journal is © The Royal Society of Chemistry 2021

Electronic Supplementary Information (ESI)

Copper sulfide as the cation exchange template of bimetallic catalysts for CO_2 electroreduction

Contents

Synthetic strategies 2
- Materials 2
- Colloidal synthesis for Cu sulfide nanosheets (C-nano-0) 2
- Synthesis of $Cu(OH)_2$ on Cu foil 2
- Synthesis of $Cu(OH)_2$ on the carbon substrate 2
- Sulfurization for Cu sulfides on Cu foil (C-foil-x) 2
- Cation exchange for bimetallic sulfides 3
- The estimation of thickness for modified surface layer on Cu foil 3

Electrochemical measurement details 3
- Electrode preparation for nanosheet samples (C-nano-0 and CA-nano-x) 3
- Electrochemical measurements 3
- Products analysis 4

Supplemental catalyst characterization and CO_2 reduction performance 4
- $Cu(OH)_2$ on the carbon substrate 4
- $Cu(OH)_2$ on Cu foil by chemical oxidation 5
- Cu sulfide nanosheets (C-nano-0) 6
- Bimetallic sulfide nanosheets (CA-nano-x) 6
- Nanosheets anchored on carbon substrate for tests 8
- Cation exchange for bimetallic sulfides on Cu foil (CA-foil-x-y) 10
- Catalyst evolution and corresponding performance change 11

Supplemental references 13

Synthetic strategies

Materials

Potassium carbonate (99.995% metals basis), nitric acid (70%), hydrochloric acid (37%), silver nitrate (99%), sodium hydroxide (98%), ammonium hydroxide solution (30%), sodium sulfate decahydrate (99%), oleylamine (70%), hexanes (98%), ethanol (100%), copper (I) thiocyanate (99%) were purchased from Sigma-Aldrich. Carbon black was purchased from AkzoNobel. Selemion AMV anion exchange membranes were purchased from Asahi Glass Co., Ltd. Substrates used here were carbon paper (Sigracet 39 BB, FuelCellStore), carbon cloth (WOS1009, Phychemi), glassy carbon plate (type 2, Alfa Aesar), and Cu foil (99.999%, Alfa Aesar). 18.2 MΩ deionized (DI) water was produced by a Millipore system. The solutions mentioned in this paper are aqueous if there is no specific description. All chemicals were used without further purification. Carbon dioxide (99.999%), argon (99.999%), and hydrogen (99.999%) were purchased from Praxair and used for electrochemical tests and gas chromatography.

Colloidal synthesis for Cu sulfide nanosheets (C-nano-0)

The Cu sulfide nanosheets (C-nano-0) were synthesized through the process modified from a previously reported recipe.[1,2] Specifically, 257 mg copper(I) thiocyanate (CuSCN) was dispersed in 25 mL oleylamine (OAM). The mixture was degassed at room temperature for 10 min. The flask was then heated to 100°C (5°C min^{-1}), then degassed for another 10 min. After that, N_2 was added to the flask before the temperature was raised at the speed of 5°C min^{-1} until 240°C, and held for another 30 min. The synthesized nanosheets were then transferred with hexanes from the flask to centrifuge tubes and were cleaned with ethanol (added to the hexanes dispersion for centrifuge), and hexanes (for redispersion of nanosheets with sonication) 3 times (8000 rpm, 5 min). The nanosheets were then dispersed in hexanes, and the concentration of the hexanes dispersion was determined by weighing the mass of a small volume of the dispersion after hexanes evaporated. The dispersion was sonicated for 10 min before use.

Synthesis of $Cu(OH)_2$ on Cu foil

Two methods have been used to grow $Cu(OH)_2$ on Cu foil: (1) electro-oxidation[3] and (2) chemical oxidation.[4] Cu foil was first cleaned and etched by sonication in 4 M HCl for 15 min, rinsed with DI water, and dried under N_2 flow. The electro-oxidation was conducted in a two-electrode system, where the Cu foil was used as the working electrode, Pt as the counter electrode, and 1 M NaOH as the electrolyte. For chemical oxidation, the Cu foil was immersed into 0.33 M ammonium hydroxide for 3 to 24 h, rinsed with DI water, and dried under N_2 flow.

Synthesis of $Cu(OH)_2$ on the carbon substrate

The $Cu(OH)_2$ on the carbon substrate was prepared with a modified electro-oxidation method.[3] The carbon paper or carbon cloth was first cleaned with sonication, 15 min in 1 M HNO_3 followed by 15 min in DI water, then dried with N_2 flow. 50 nm layer of Cu was deposited on each side using an e-beam evaporation system (Angstrom Engineering Inc., Nexdep). The region other than the testing area was sheltered to block the deposition. The electro-oxidation method used is similar to that used for Cu foil.

Sulfurization for Cu sulfides on Cu foil (C-foil-x)

The $Cu(OH)_2$ electrode was cut into a 2 cm × 2 cm square and was immersed in 0.1 M Na_2S at 90°C for 12 h. The electrode was taken out from the Teflon reactor after the temperature decreased to room temperature. The electrode was dried under N_2 flow after thoroughly rinsing with DI water.

Cation exchange for bimetallic sulfides

The bimetallic sulfide nanosheets (CA-nano-x) were obtained by a cation exchange method.[3] The previously prepared hexanes dispersion of C-nano-0 was used for cation exchange and the volume added was calculated from the concentration. Typically, the dispersion containing 30 mg C-nano-0 was added to 7 mL OAM, Ag precursor silver nitrate ($AgNO_3$) was then added to the mixture (Table 1 in the main text for sample nomenclature). The solution was degassed and distilled (hexanes) at room temperature for 20 min, the temperature was then increased to 50°C, and N_2 was added back to the flask. The flask was then kept at 50 °C for another 30 min. The nanosheets were then cleaned with the same process as C-nano-0. For bimetallic sulfides on Cu foil (CA-foil-x-y), the $AgNO_3$ was first added to 7 mL OAM in a small petri-dish, N_2 gas was bubbled into the solution for 10 min before the temperature is raised to 50°C, the electrode was then put into the dish, and shake for another 30 min. The electrode was then taken out and sonicated in hexanes for 15 min, and ethanol for another 15 min, and dried under N_2 flow.

The estimation of thickness for modified surface layer on Cu foil

The number was estimated assuming all the electrons in electro-oxidation contributed to the formation of $Cu(OH)_2$, specifically, one atom of Cu^+ achieved from Cu counts one electron. The calculation here is for CA-foil-20-y/C-foil-20, with the electro-oxidation at a current density of 20 mA cm^{-2} for 30 min to obtain $Cu(OH)_2$ layer, based on the parameters below.

The density of Cu: $8.92\ \mathrm{g\ cm^{-3}}$, molar mass: $63.546\ \mathrm{g\ mol^{-1}}$.

Thus, $\frac{63.546\ \mathrm{g\ mol^{-1}}}{8.92\ \mathrm{g\ cm^{-3}}} = 7.124\ \mathrm{cm^3\ mol^{-1}}$

Total electrons consumed:

$$20\ \mathrm{mA\ cm^{-2}} \times\ 30\ \mathrm{min}\ \times 2\ \mathrm{cm} \times 2\ \mathrm{cm} = 144\ \mathrm{C} =\ 8.99 \times 10^{20}\ \mathrm{electrons}\ = 0.00149\ \mathrm{mol\ electrons}.$$

That means the volume of Cu modified:

$$0.00149\ \mathrm{mol} \times 7.124\ \mathrm{cm^3\ mol^{-1}} =\ 0.0106\ \mathrm{cm^3}$$

The thickness was obtained by the volume divided by the total area of both sides of the Cu foil. $\frac{0.0106\ \mathrm{cm^3}}{8\ \mathrm{cm^2}} \approx\ 13.25\ \mathrm{\mu m}$

Electrochemical measurement details

Electrode preparation for nanosheet samples (C-nano-0 and CA-nano-x)

The nanosheet samples were first mixed with carbon black (mass ratio, 1:1) and then drop cast on the glassy carbon plate. To anchor catalysts on carbon, the carbon black was first dispersed in hexanes and sonicated for 30 min to reach a homogenous suspension. The catalyst dispersion was then added in drops to the carbon suspension under sonication. The mixed solution was sonicated for another 30 min. The catalyst was then washed and centrifuged with ethanol (8000 rpm, 5 min) 3 times. The catalyst-carbon mix was then ground before use. For catalyst ink, typically, 60 mL of Nafion solution (5 wt.%, DuPont Co.) was added into 1 mL catalyst suspension that contains 3 mg catalyst (volumetric ratio of ethanol/water is 3:1). The electrode was dried overnight at room temperature. The absence of ethanol detected at ~−0.2 V vs. RHE demonstrates no ethanol contaminants exist before CO_2 reduction.

Electrochemical measurements

The Biologic SP-300 potentiostat was used for electrochemical analysis. The CO_2 reduction was carried out in 0.05 M K_2CO_3 using a three-electrode system with Ag/AgCl (saturated KCl) as reference and Pt plate as the counter electrode. The working electrode and counter electrode with an area of 0.785 cm^2 were placed facing each other in the cathodic and anodic chamber (2.4 mL electrolyte in each), respectively, separated by the anion exchange membrane. The CO_2 gas was purged at 5 sccm to the cathodic chamber, and the test started after 15 min purging when the solution was saturated with CO_2.

The potential was converted to the RHE scale using the following equation:

$$E \text{ vs. RHE} = E \text{ vs. Ag/AgCl} + 0.197 \text{ V} + 0.0591 \times pH$$

85% of solution resistance was corrected during the electrochemical experiments.

Products analysis

All gas products including H_2, CO, methane, ethylene, ethane, were detected by MG#3 Gas Chromatography from SRI Instruments, equipped with a 12" long HaySep D column and Ar as the carrier gas. H_2 was detected by a TCD detector, others were detected by an FID equipped with a methanizer.[5] The outlet of the electrochemical cell was linked to the GC during the whole run, and for a typical 1.5 h test, 6 results can be obtained, and the average of the last 5 runs gives the gas product results. The GC was calibrated with 3 calibration gas cylinders with different concentrations.

All liquid products were detected by NMR (Bruker Avance III 500 MHz). Typically, 700 mL of electrolyte, containing CO_2 reduction products, were mixed with 35 mL internal standard solution: 10 mM dimethyl sulfoxide (DMSO) and 50 mM phenol in D_2O. The products were analyzed mainly according to a well-organized table in the reference.[6]

The Faradaic efficiency (FE) for each product was calculated by $FE = znF/Q$, where z is the number of exchanged electrons, n is the moles of the product produces, F is the Faraday constant, Q is the charge passed.

Supplemental catalyst characterization and CO_2 reduction performance

$Cu(OH)_2$ on the carbon substrate

$Cu(OH)_2$ grown on the carbon paper have a nanosheet structure, however not uniformly distributed because of the shadowing effect during Cu deposition, the randomly aligned nanofibers, and the uneven soaking of the electrolyte of carbon paper. The electro-oxidation process was conducted in 1 M NaOH, at the current density of 10 mA cm^{-2} for 5 min.

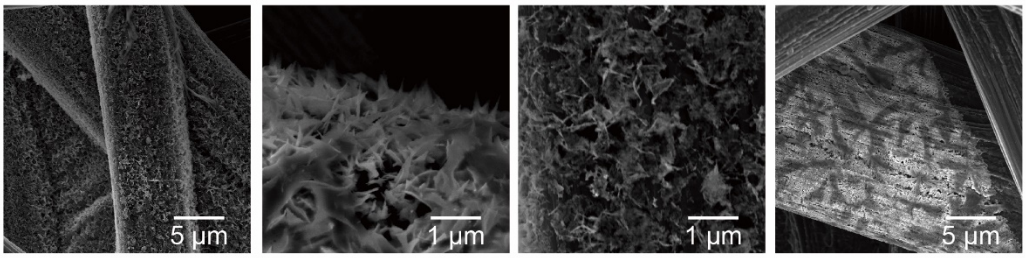

Figure S1 SEM images of $Cu(OH)_2$ on the carbon paper obtained by the electro-oxidation method.

To have more control on the $Cu(OH)_2$ distribution, carbon cloth composed with aligned nanofibers was used as the carbon substrate. Small current density achieves large thin nanosheets, while larger current density achieves small thick sheets.

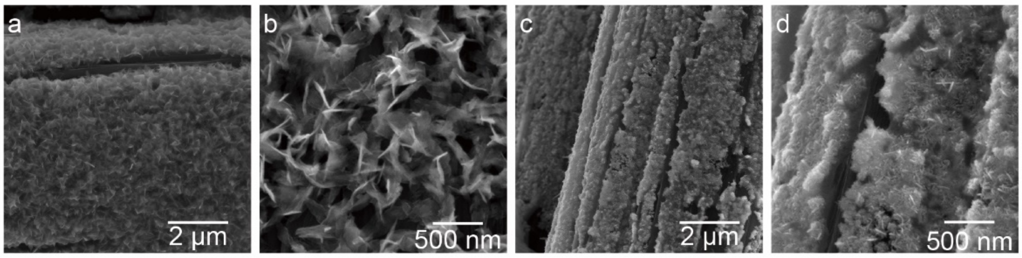

Figure S2 SEM images of $Cu(OH)_2$ on the carbon cloth obtained by the electro-oxidation method. (a, b) were obtained with the current density of 1.25 mA cm^{-2} for 10 min; (c, d) were obtained with the current density of 2.5 mA cm^{-2} for 10 min.

However, after the sulfurization process, most of the Cu-containing nanosheets detached from the carbon fibers because of the weak binding between the deposited copper and the carbon fibers as well as the harsh sulfurization condition.

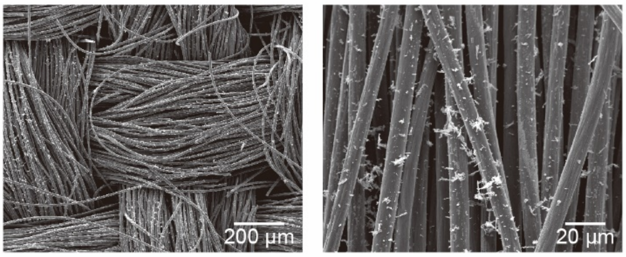

Figure S3 $Cu(OH)_2$ on carbon cloth after sulfurization.

$Cu(OH)_2$ on Cu foil by chemical oxidation

Considering the detachment of Cu-containing nanosheets from the carbon substrate during the sulfurization, chemical oxidation directly on the Cu foil substrate was tried.

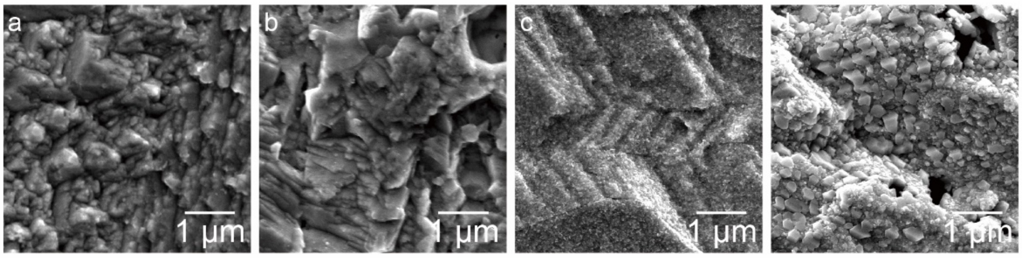

Figure S4 $Cu(OH)_2$ grown on Cu foil by chemical oxidation. The deposition time from (a) to (d) is 3 h, 6 h, 9 h, and 24 h, respectively.

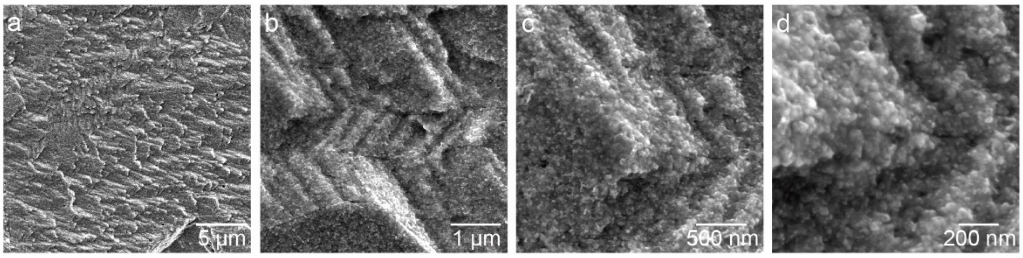

Figure S5 $Cu(OH)_2$ grown on Cu foil after chemical oxidation for 9 h.

After sulfurization, the Cu sulfide nanosheets can be clearly seen from the SEM images; however, the nanosheets tend to gather instead of uniformly distribute through the whole surface.

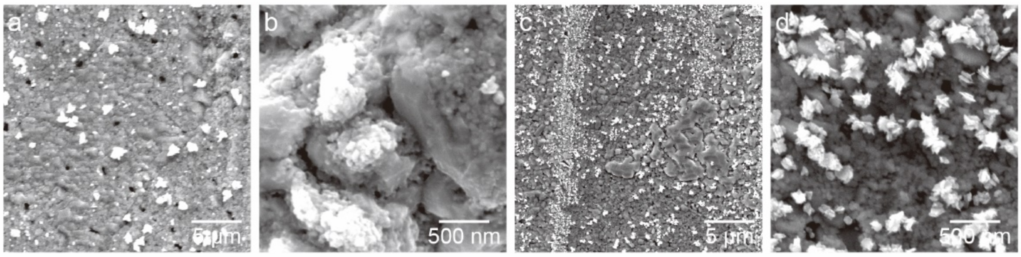

Figure S6 Cu sulfides on Cu foil obtained by hydrothermal sulfurization from chemical oxidation derived $Cu(OH)_2$. (a, b) were from $Cu(OH)_2$ obtained by 9 h chemical oxidation. (c, d) were from 24 h chemical oxidation.

Cu sulfide nanosheets (C-nano-0)

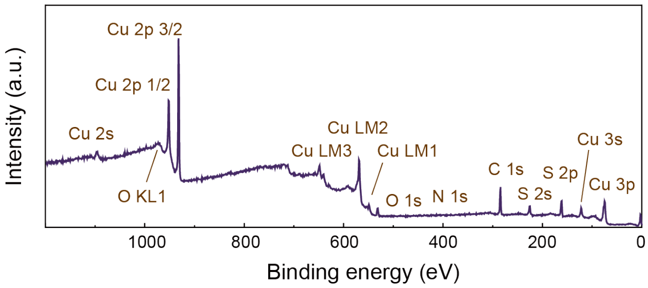

Figure S7 Full XPS spectrum of C-nano-0. The S/Cu atomic ratio (0.67) was determined from the Cu 2p and S 2p peak areas with appropriate sensitivity factors

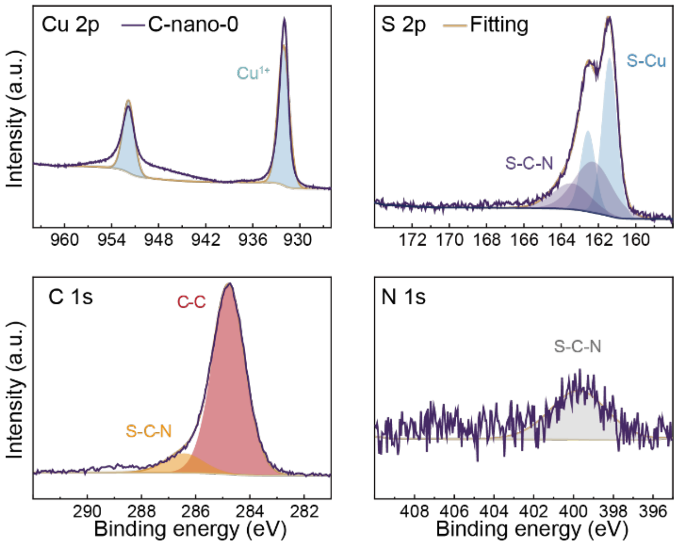

Figure S8 High-resolution XPS spectra of Cu 2p, S 2p, C 1s, and N 1s for C-nano-0. The existence of N, the doublet at 163 eV in the S_{2p} spectrum, and the peak at 286 eV in the C_{1s} spectrum indicate a small amount of residual ligand -SCN from the precursor CuSCN.

Bimetallic sulfide nanosheets (CA-nano-x)

SEM images and the Ag/Cu mass ratio obtained from ICP-MS data demonstrated successful replacement of Cu with Ag during the cation exchange process (Figures S9–12). For nanosheets with ultra-high Ag concentration (CA-nano-4), the regular hexagonal shape distorted to irregular ones with the nanosheet structure remains. HRTEM images and corresponding FFT patterns of basal planes show that crystal structure becomes more complex after cation exchange.

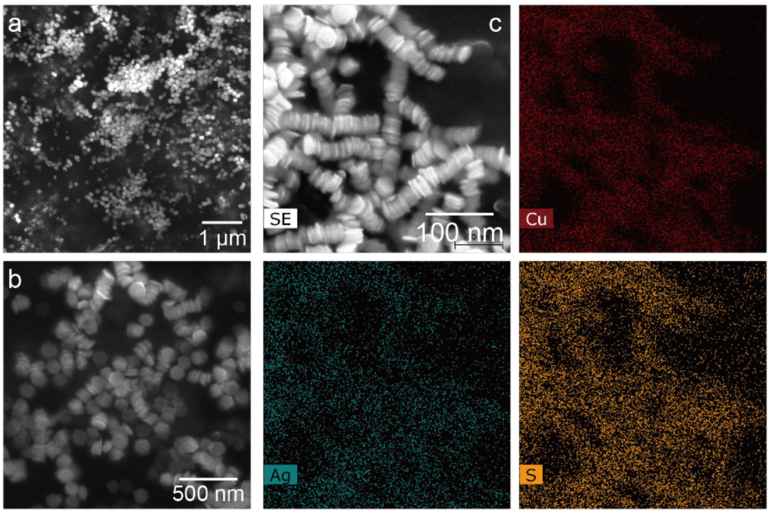

Figure S9 Morphology and composition of CA-nano-1. Ag/Cu mass ratio is 0.3. (a, b) SEM images. (c) SEM images with elemental mapping.

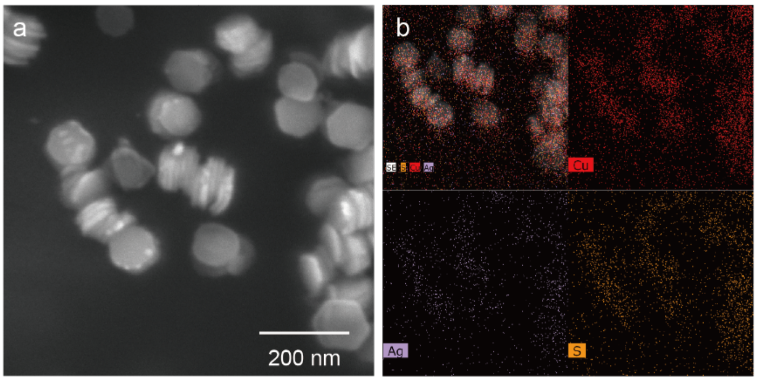

Figure S10 Morphology and composition of CA-nano-2. Ag/Cu mass ratio is 0.8. SEM image (a) with elemental mapping (b).

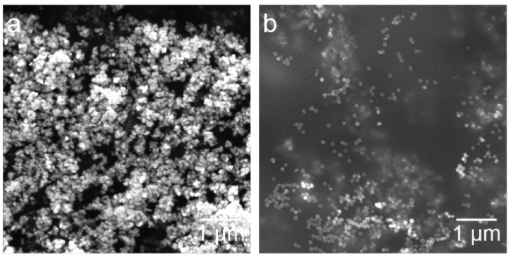

Figure S11 Morphology and composition of CA-nano-3. Ag/Cu mass ratio is 2.3. (a, b) SEM images.

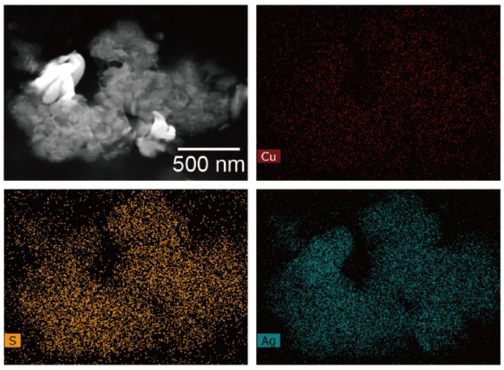

Figure S12 Morphology and composition of CA-nano-4. Ag/Cu ratio is 25. SEM image with elemental mapping.

Table S1 The Ag/Cu mass ratios were quantified by inductively coupled plasma mass spectroscopy (7900 ICP−MS, Agilent) using the He mode. The internal standard was Ge or Rh selected based on its first ionization potential and M/Z as compared to Cu or Ag, respectively.

Label	Ag (ppb)	Cu (ppb)	Ag/Cu mass ratio
CA-nano-1	16.5140223	48.59833275	0.33980636
CA-nano-2	112.2584741	140.277679	0.80025899
CA-nano-3	465.7222782	204.32315	2.27934172
CA-nano-4	155.4151727	6.22519108	24.9655265

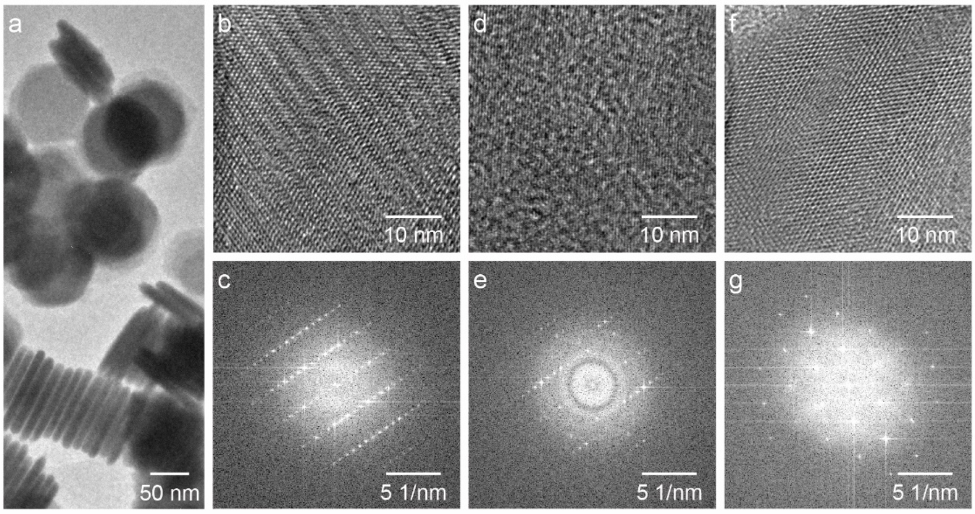

Figure S13 Characterization of CA-nano-2 nanosheets. (a) TEM images. HRTEM images (b, d, f) and corresponding FFT patterns (c, e, g) of basal planes show each nanosheet may contain several small crystals with completely different exposed facets or even composition, although the shape does not vary much.

Nanosheets anchored on carbon substrate for tests

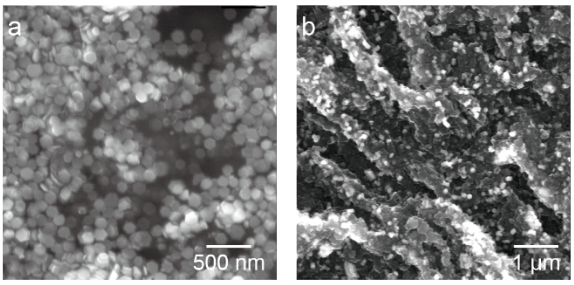

Figure S14 Morphology of C-nano-0 nanosheets and their uniform distribution on porous carbon. (a) SEM image for C-nano-0 nanosheets. (b) SEM image for C-nano-0 dispersed on carbon black.

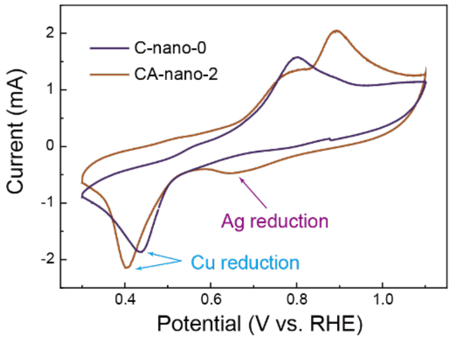

Figure S15 CV plot for CA-nano-2 compared with C-nano-0 control. The curves suggest both Cu and Ag are catalytically active for bimetallic nanosheets. Sweeping speed: 10 mV s^{-1}.

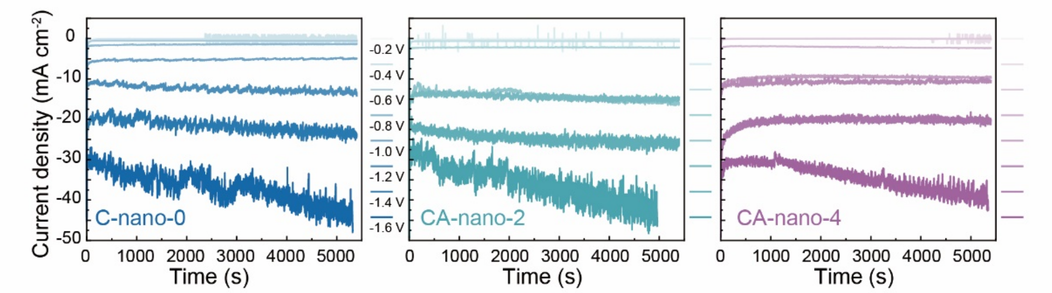

Figure S16 Chronoamperometry (CA) plot of sulfide nanosheet with applied potential in the range from −0.2 V to −1.6 V vs. RHE.

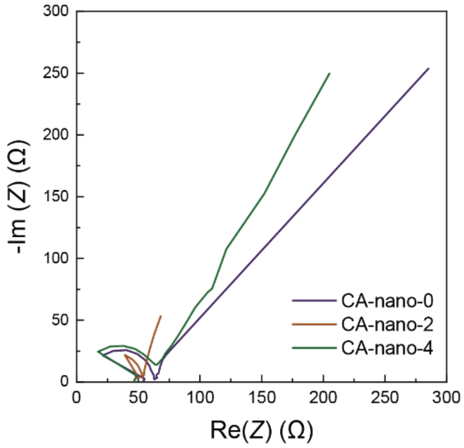

Figure S17 Electrochemical impedance spectra (EIS) for sulfide nanosheets conducted at the open-circuit voltage (OCV).

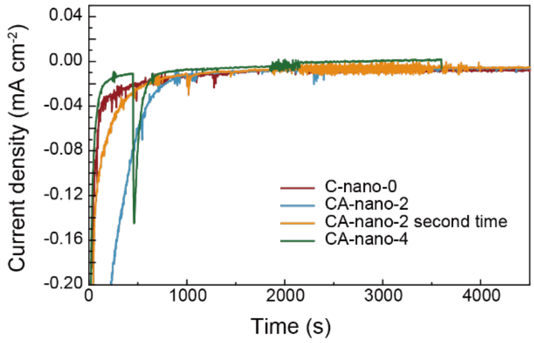

Figure S18 Current density for nanosheet samples at −0.2 V vs. RHE. CA-nano-2 second time represents the electrode tested at −0.2 V vs. RHE after previously tested at more negative potentials.

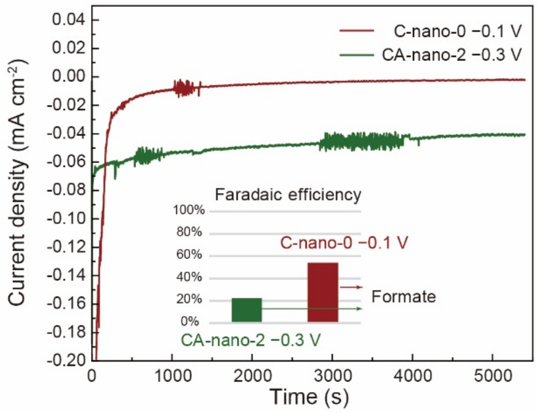

Figure S19 Current density for sulfide nanosheets at small negative potentials. Inset: Faradaic efficiency plot, the only product detected was formate, although the Faradaic efficiency may not be the accurate number due to testing limit.

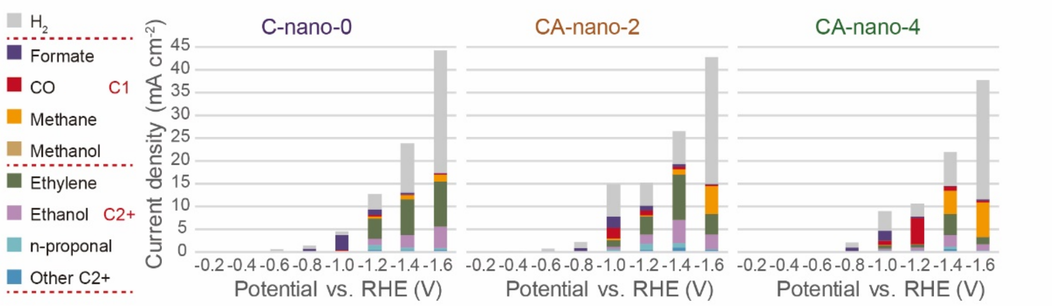

Figure S20 Partial current density bar plots for sulfide nanosheets.

Cation exchange for bimetallic sulfides on Cu foil (CA-foil-x-y)

Ag concentration can be well controlled by the amount of Ag precursor $AgNO_3$ added. With a low concentration in OAM, Cu can be uniformly exchanged by Ag throughout the electrode. As the precursor increased, Ag nucleation started to appear, see the bright spot in Figure S21c. Since the Cu foil can also contribute to the Cu concentration in the EDS result, no quantitative Ag/Cu mass ratio is provided. However, the comparison between areas of Cu and Ag peaks in EDS results still demonstrates the control.

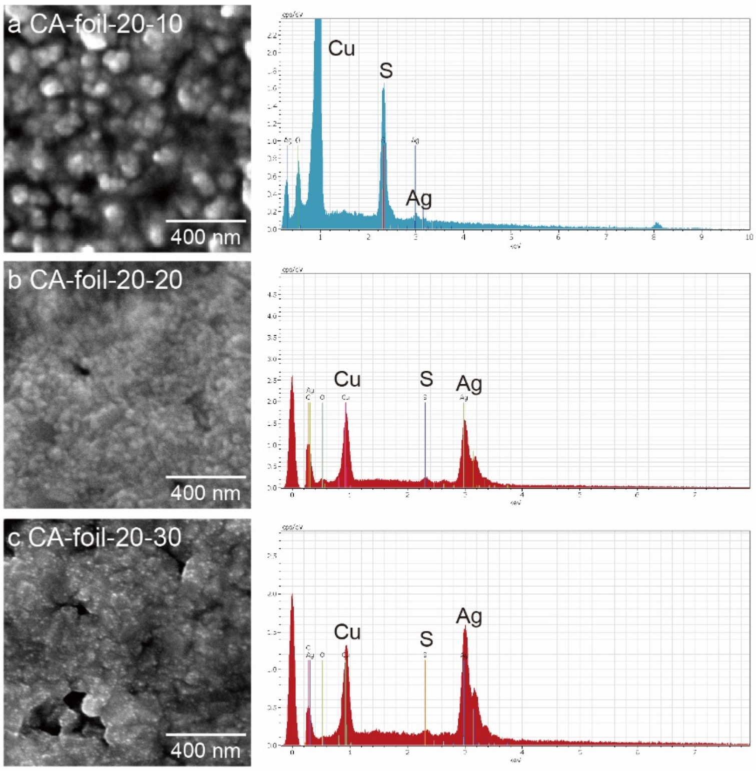

Figure S21 Morphology and elemental concentration of CA-foil-x-y. The Ag precursor $AgNO_3$ added for (a) to (c) was 10, 20, 30 mg, respectively.

The extra silver added during cation exchange resulted in Ag nucleation on the surface.

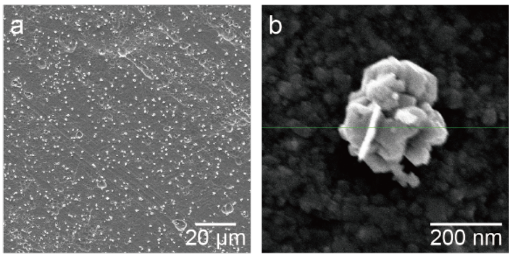

Figure S22 Morphology of CA-foil-20-40 as-synthesized.

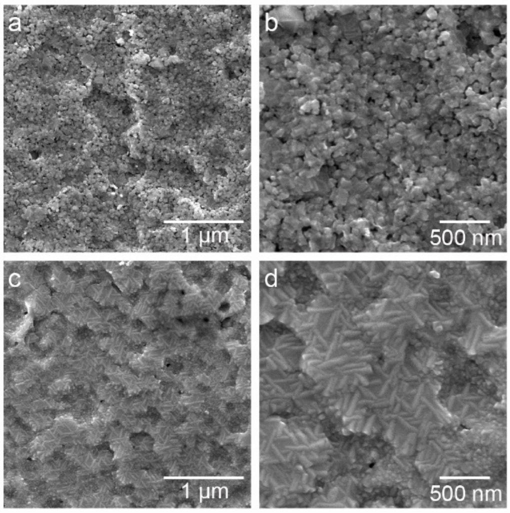

Figure S23 Morphology of Cu sulfides on Cu foil C-foil-40 (a, b), and its cation exchanged counterpart CA-foil-40-40 (c, d) when the original particle size was large (100 nm).

Catalyst evolution and corresponding performance change

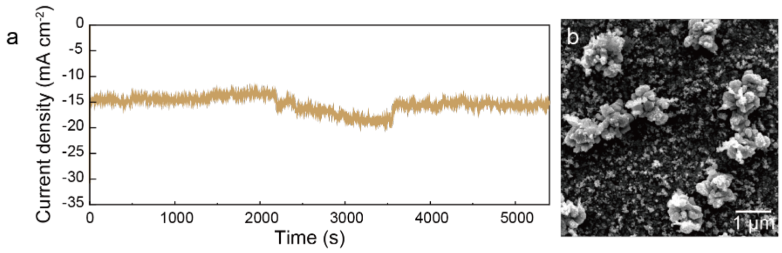

Figure S24 (a) Current density plot of CA-foil-20-40 at −1.2 V vs. RHE. (b) Morphology of CA-foil-20-40 after CO_2 reduction.

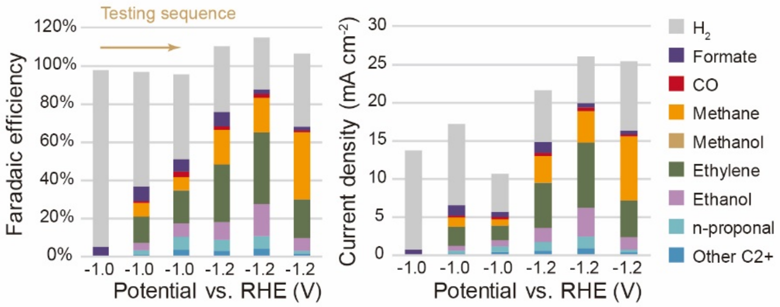

Figure S25 Product profile changes with the testing sequence. FE and current density bar plots for CA-foil-20-40. The columns in the plot represent different tests with the testing sequence from left to right.

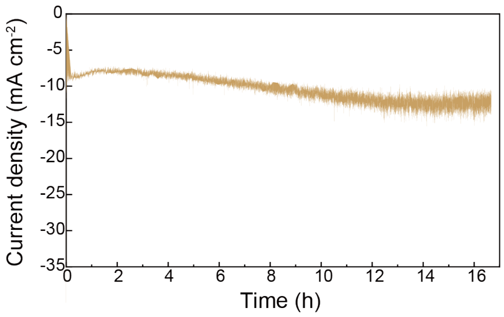

Figure S26 Current density plot of CA-foil-20-40 for 16 h CO_2 reduction at −1.0 V vs. RHE.

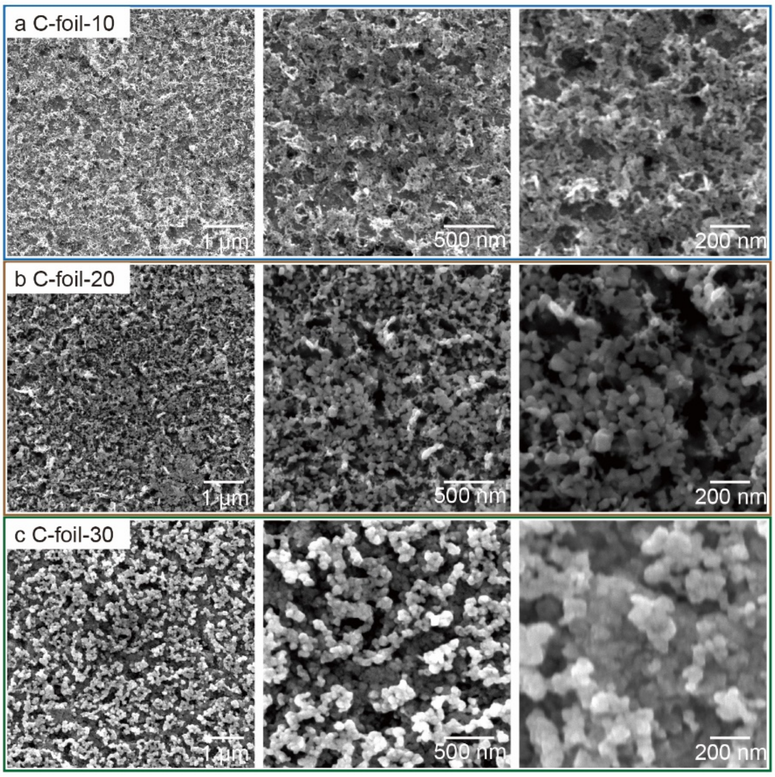

Figure S27 Morphology of C-foil-x after CO_2 reduction.

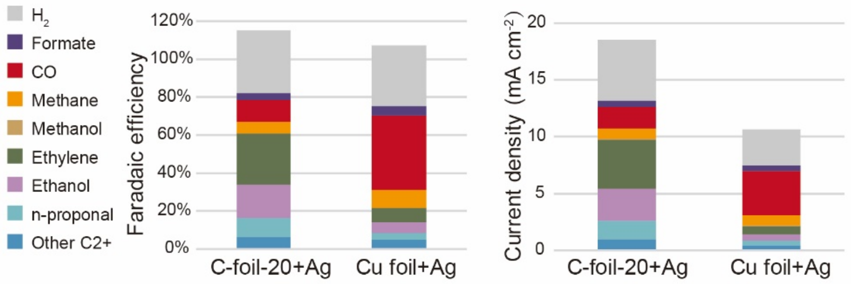

Figure S28 CO_2 reduction performance at −1.1 V vs. RHE (FE and current density bar plots) for C-foil-20 and Cu foil with 100 nm deposited Ag layer on top.

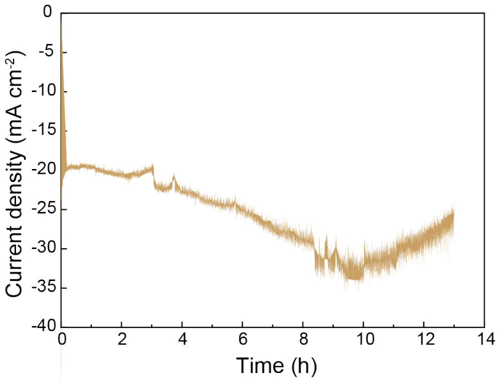

Figure S29 Current density plot of CA-nano-4 for 13 h CO_2 reduction at −1.4 V vs. RHE.

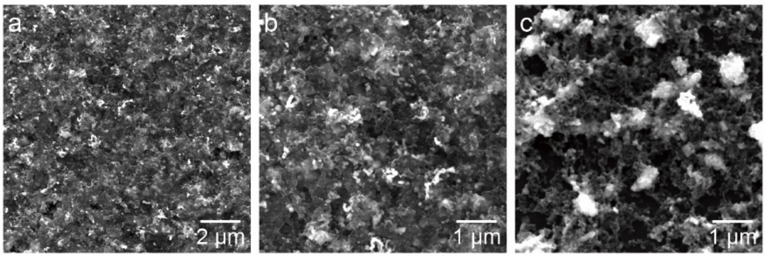

Figure S30 Morphology of nanosheets samples anchored on carbon after CO_2 reduction. SEM images for CA-nano-4 (a, b) and C-nano-0 (c) after CO_2 reduction.

Supplemental references

(1) Kim, T.; Park, J.; Hong, Y.; Oh, A.; Baik, H.; Lee, K. Janus to Core-Shell to Janus: Facile Cation Movement in $Cu_{2-x}S/Ag_2S$ Hexagonal Nanoplates Induced by Surface Strain Control. *ACS Nano* **2019**, *13*, 11834–11842.

(2) Park, J.; Park, J.; Lee, J.; Oh, A.; Baik, H.; Lee, K. Janus Nanoparticle Structural Motif Control via Asymmetric Cation Exchange in Edge-Protected $Cu_{1.81}S@Ir_xS_y$ Hexagonal Nanoplates. *ACS Nano* **2018**, *12*, 7996–8005.

(3) Deng, S.; Shen, Y.; Xie, D.; Lu, Y.; Yu, X.; Yang, L.; Wang, X.; Xia, X.; Tu, J. Directional Construction of Cu_2S Branch Arrays for Advanced Oxygen Evolution Reaction. *J. Energy Chem.* **2019**, *39*, 61–67.

(4) Wen, X.; Zhang, W.; Yang, S. Synthesis of $Cu(OH)_2$ and CuO Nanoribbon Arrays on a Copper Surface. *Langmuir* **2003**, *19*, 5898–5903.

(5) Lum, Y.; Cheng, T.; Goddard, W. A.; Ager, J. W. Electrochemical CO Reduction Builds Solvent Water into Oxygenate Products. *J. Am. Chem. Soc.* **2018**, *140*, 9337–9340.

(6) Kuhl, K. P.; Cave, E. R.; Abram, D. N.; Jaramillo, T. F. New Insights into the Electrochemical Reduction of Carbon Dioxide on Metallic Copper Surfaces. *Energy Environ. Sci.* **2012**, *5*, 7050.
